# Editorial: Biomolecular approaches to trichomoniasis: epidemiology, diagnosis, and treatment

**DOI:** 10.3389/fpara.2025.1691804

**Published:** 2025-10-02

**Authors:** Alexandra Ibáñez-Escribano, Tiana Tasca, Valentina Margarita

**Affiliations:** ^1^ Department of Microbiology and Parasitology, Complutense University of Madrid, Madrid, Spain; ^2^ Faculty of Pharmacy and Centre of Biotechnology, Federal University of Rio Grande do Sul, Porto Alegre, Brazil; ^3^ Department of Biomedical Sciences, University of Sassari, Sassari, Italy

**Keywords:** *Trichomonas vaginalis*, biomarker, therapy, *in-vitro* model, natural products, endobionts

Trichomoniasis is a significant public health concern, particularly due to its adverse effects during pregnancy and its established role in facilitating the acquisition of other sexually transmitted infections (STIs), as well as in the development of prostate and cervical cancers. Despite being classified by the Centers for Disease Control and Prevention as a neglected parasitic infection, recent molecular and biological advancements have greatly enhanced our understanding of this protozoan. The latest World Health Organization (WHO) report on global health sector strategies on STIs (2022–2030), which aims to reduce new STI cases by 50% by 2030, highlights the urgency of addressing this pathogen, particularly in the context of increasing treatment resistance. This topic brings together the latest research on *Trichomonas vaginalis*, with a focus on its virulence, the identification of therapeutic targets, and the development of effective treatment strategies.

The article by Aranda-Chan et al. shows the role of trichocystatin 2 (TC-2) as a potential biomarker. TC-2 has been previously identified as one of the three endogenous inhibitors of the parasite’s cathepsin L-like cysteine-proteases. TC-2 inhibits the activity of papain and cathepsin L as well as the proteolytic activity of *T. vaginalis* protease-resistant extracts and was even able to protect HeLa cell monolayers from cytotoxic damage caused by the parasite. The challenge shown in this work regards solving the production of multimeric aggregates when TC-2 is recombinantly expressed in *E. coli*, which could impair its stability and production on a larger scale and with a longer production time. Considering that TC-2 possesses five cysteines, including four located at the N terminus, these cysteines are proposed to promote the formation of recombinant TC-2 multimers. In this study, a recombinant TC-2 mutant was expressed, purified, characterized, and compared with the recombinant wild-type TC-2 protein. The results showed that the four cysteines located in the N-terminal region are responsible for aggregation, and their deletion affected the interaction of TC-2 with papain without affecting its inhibitory activity on homologous target proteases that are crucial for *T. vaginalis* virulence, suggesting TC-2 as a potential therapeutic target.

Likewise, continuing with the study of certain molecules of the parasite as potential biomarkers, the research conducted by Euceda-Padilla et al. was focused on the cysteine peptidases TvLEGU-1 and TvLEGU-2 present in the vaginal secretions of infected women. Cysteine peptidases are an extensive group of proteins, some of them contributing to the parasite’s pathogenesis. Considering the glucose fluctuations observed in the vagina and during trichomoniasis, the effect of glucose on these two biomarkers was evaluated *in vitro*. The results suggest that both TvLEGU-1 and TvLEGU-2 are secreted in different vesicles in a time-dependent manner and play an important role during *T. vaginalis* infection under different glucose conditions.

The *in-vitro* co-culture model of *Trichomonas vaginalis*, *Candida albicans*, and *Lactobacillus crispatus* by Cardoso et al. was published in this Research Topic, demonstrating a system for assessing antimicrobial activity and microorganism interactions in vaginitis. In this study, the authors established an *in-vitro* co-culture of *Trichomonas vaginalis*, *Candida albicans*, and *Lactobacillus crispatus* to simulate the vaginal microenvironment at the site of infection. Man-Rogosa-Sharpe broth (MRS) medium was chosen for the co-culture, with initial cell densities determined as trophozoites counted in a hemocytometer and colony-forming units (CFUs) on selective agar for *C. albicans* and *L. crispatus*. The co-culture system demonstrated lower MIC values for standard treatments such as metronidazole and fluconazole. Furthermore, the triple co-culture increased the *T. vaginalis* cytotoxicity to vaginal cells and erythrocytes while significantly inhibiting both biofilm formation and metabolic activity of *C. albicans*, as well as its yeast-to-hyphae transition. This co-culture system is a valuable tool for evaluating the antimicrobial efficacy of novel compounds against vaginitis pathogens and for studying interactions within the vaginal microenvironment.

In the context of exploring novel pharmacological alternatives, Margarita et al. investigated the trichomonacidal activity of essential oils (EO) from *Cymbopogon citratus, Citrus grandis*, and *Mentha arvensis*, as well as their effects on the two endobiont bacteria harbored by *Trichomonas vaginalis*: *Mycoplasma hominis* and *Candidatus* Mycoplasma girerdii. The study assessed the effect of these extracts against 30 *T. vaginalis* isolates, both with and without mycoplasma endobionts, and with varying levels of metronidazole susceptibility. Among the tested EO, *C. citratus* displayed the most consistent activity across all isolates. Importantly, none of the extracts exhibited unspecific cytotoxicity on mammalian cells or disrupted the vaginal microbiota. These findings highlight the potential of medicinal plants as valuable sources of effective and safe alternatives against *T. vaginalis* while preserving vaginal health.

In summary, recent advances have highlighted promising directions for the diagnosis and treatment of trichomoniasis. Novel biomarkers such as TC-2 and TvLEGU-1 and TvLEGU-2 provide new insights into parasite virulence and host interactions. TC-2 can be used as a potential therapeutic target since it was identified as a regulator of cysteine-protease activity, protecting host cells from *T. vaginalis* cytotoxicity. TvLEGU-1 and TvLEGU-2, previously characterized as immunogenic and found in the vaginal secretions of patients with trichomoniasis, were also secreted *in vitro* under the influence of glucose in a time-dependent manner and show proteolytic activity. These findings support their involvement in parasite pathogenesis, reinforcing their diagnostic and therapeutic relevance.

Host-pathogen-microbiota interactions were also investigated through an innovative *in-vitro* co-culture model involving *T. vaginalis*, *C. albicans*, and *L. crispatus*, mimicking the vaginal environment. The system demonstrated lower MIC values for standard treatments compared to monocultures of *T. vaginalis* and *C. albicans*, with an increment of protozoan cytotoxicity against vaginal cells and erythrocytes. Inhibition of both biofilm formation and metabolic activity of *C. albicans* together with yeast-to-hyphae transition was also observed. These data support and underscore the development of an *in-vitro* co-culture system for testing antimicrobial efficacy under physiologically relevant conditions.

In parallel, natural product–based strategies, particularly the use of essential oils, were evaluated as alternative treatments for trichomoniasis. The novel of this research was assessing the influence of *M. hominis* and *Ca*. M. girerdii on *T. vaginalis* susceptibility to essential oils. All three essential oils exhibited effective antitrichomonal activity, with *C. citratus* oil exhibiting the strongest inhibitory effect on *T. vaginalis*, including strains harboring bacterial symbionts, and maintaining safety for host cells and the vaginal microbiota.

Collectively, these findings emphasize the importance of integrating molecular biomarkers, advanced experimental models, and novel therapeutic approaches to advance future strategies against *T. vaginalis.*

